# Supplementary material for: Reorganization of brain structural networks in aging: A longitudinal study
Source: J Neurosci Res. 2021 Feb 2;99(5):1354–76. doi: 10.1002/jnr.24795 (PMC8248023; doi:10.1002/jnr.24795)
Supplement: Supplementary file 3 — FIGURE S1 Correlation between head‐motion relative displacement values and age for all subjects and both timepoints. Head‐motion displacement values were extracted using FSL tools and averaged across all volumes acquired for one subject. Correlation is not significant (r = 0.019, p = 0.85) meaning that age is not associated with head‐motion FIGURE S2 Comparison of head‐motion relative displacement values between timepoints. A paired t‐test was performed, and it was not significant (p = 0.95) meaning that head‐motion values did not differ between timepoints FIGURE S3 Percentage of connections lost in each subject when applying consistency‐based thresholding. Percentage is calculated as the proportion of connections removed in the subject SC matrix relative to the total number of connections removed in the group consistency mask. Plot on the left illustrates results for timepoint 1 and on the right, results for timepoint 2 FIGURE S4 Frequency distribution for the connection strength of the links removed when applying consistency‐based thresholding. Plot on the left illustrates results for timepoint 1 and on the right, results for timepoint 3 FIGURE S5 Percentage of connections that were present in the group consistency mask but were not present in all subjects’ SC matrices. Percentage is calculated as the proportion of connections not present in the subject SC matrix relative to the total number of connections in the group consistency mask. Plot on the left illustrates results for timepoint 1 and on the right, results for timepoint 2 FIGURE S6 Frequency distribution for the connection strength of the links from the group consistency mask not present in all subjects, when applying consistency‐based thresholding. Plot on the left illustrates results for timepoint 1 and on the right, results for timepoint 3 FIGURE S7 Consistent signatures of SC for M1 and M2 timepoints. Left panel shows intra‐timepoint consistency measured as the association between individual SC signatu [file JNR-99-1354-s001.docx]

**Reorganization of brain structural networks in aging: a longitudinal study**

Ana Coelho^1,2,3^, Henrique M. Fernandes^4,5^, Ricardo Magalhães^1,2,3^, Pedro S. Moreira^1,2,3^, Paulo Marques^1,2,3^, José M. Soares^1,2,3^, Liliana Amorim^1,2,3^, Carlos Portugal-Nunes^1,2,3^, Teresa Castanho^1,2,3^, Nadine Correia Santos^1,2,3^, Nuno Sousa^1,2,3^

^1^Life and Health Sciences Research Institute (ICVS), School of Medicine, University of Minho, 4710-057 Braga, Portugal.

^2^ICVS/3B’s, PT Government Associate Laboratory, 4710-057 Braga/Guimarães, Portugal.

^3^Clinical Academic Center – Braga, 4710-057 Braga, Portugal.

^4^Center for Music in the Brain (MIB), Aarhus University, Aarhus, Denmark

^5^Department of Psychiatry, University of Oxford, Oxford, UK

**Author Note**

Correspondence concerning this article should be addressed to Nuno Sousa, Life and Health Sciences Research Institute (ICVS), School of Medicine, University of Minho, Campus Gualtar, 4710-057 Braga, Portugal. Tel: +351 253 604878. Fax: +351 253 604809. Email: [njcsousa@med.uminho.pt](mailto:njcsousa@med.uminho.pt)

**
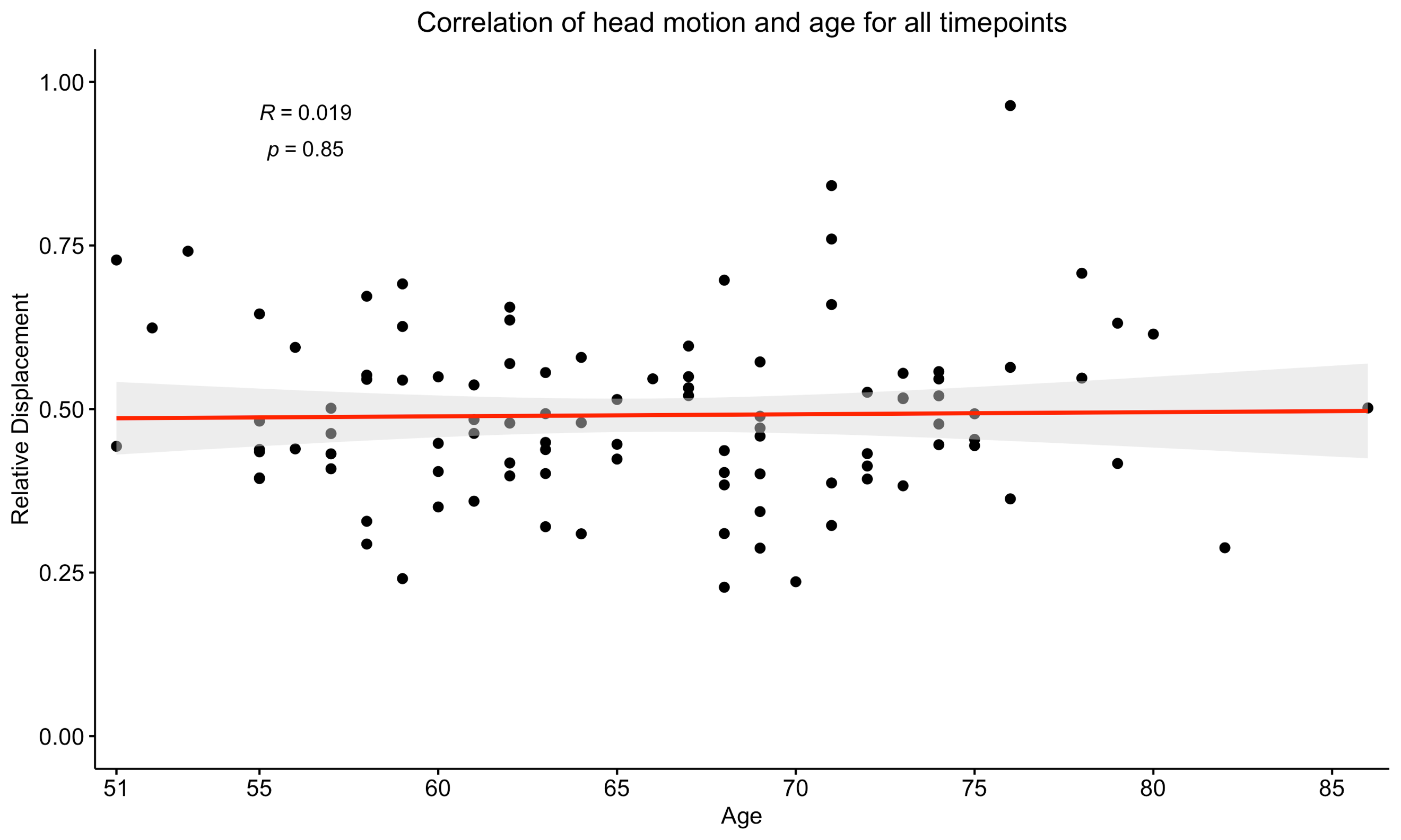
Supplementary Figures**

**Figure S1 –** Correlation between head-motion relative displacement values and age for all subjects and both timepoints. Head-motion displacement values were extracted using FSL tools and averaged across all volumes acquired for one subject. Correlation is not significant (r = 0.019, p = 0.85) meaning that age is not associated with head-motion.

**
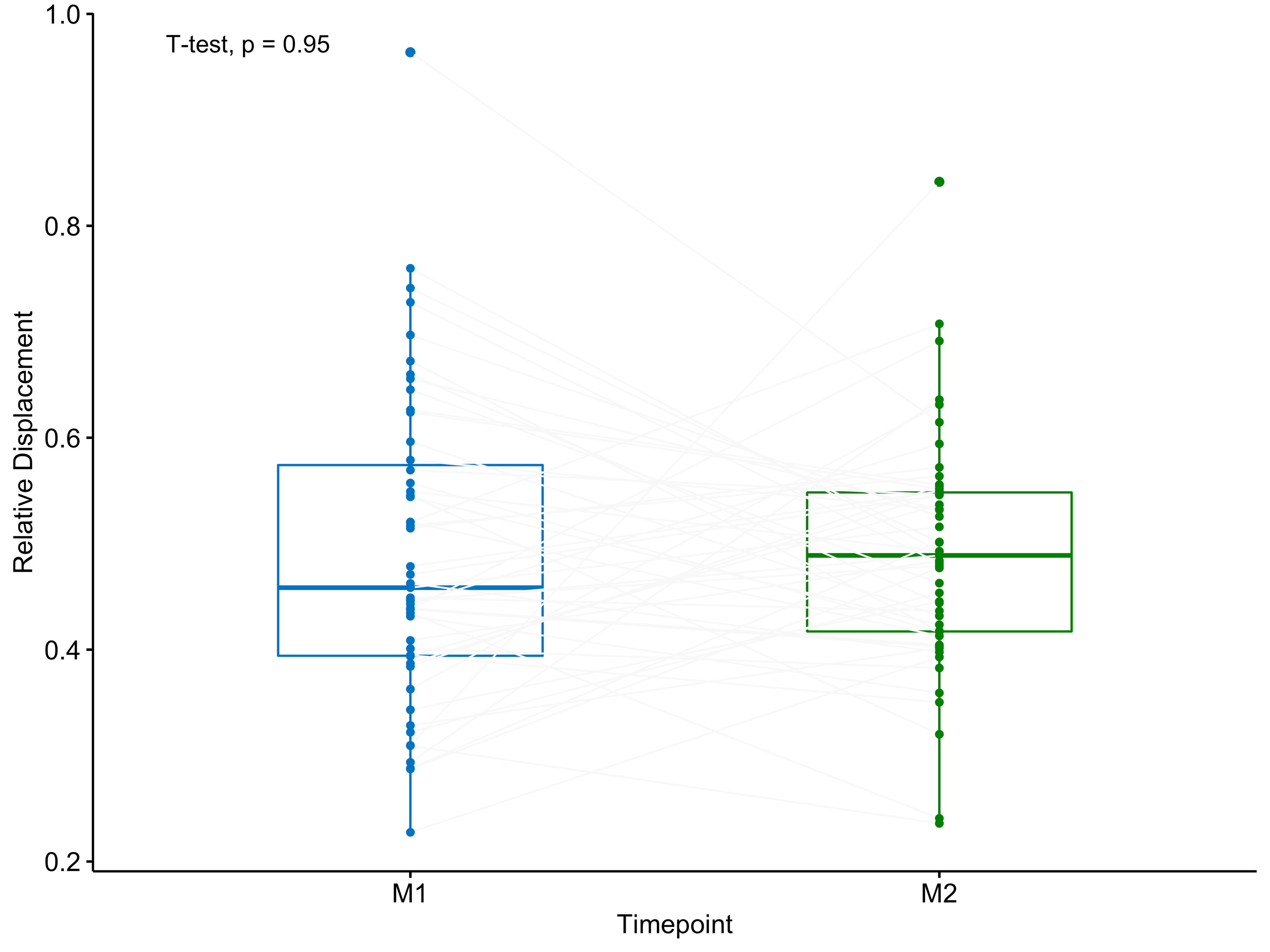
**

**Figure S2 –** Comparison of head-motion relative displacement values between timepoints. A paired t-test was performed, and it was not significant (p = 0.95) meaning that head-motion values did not differ between timepoints.


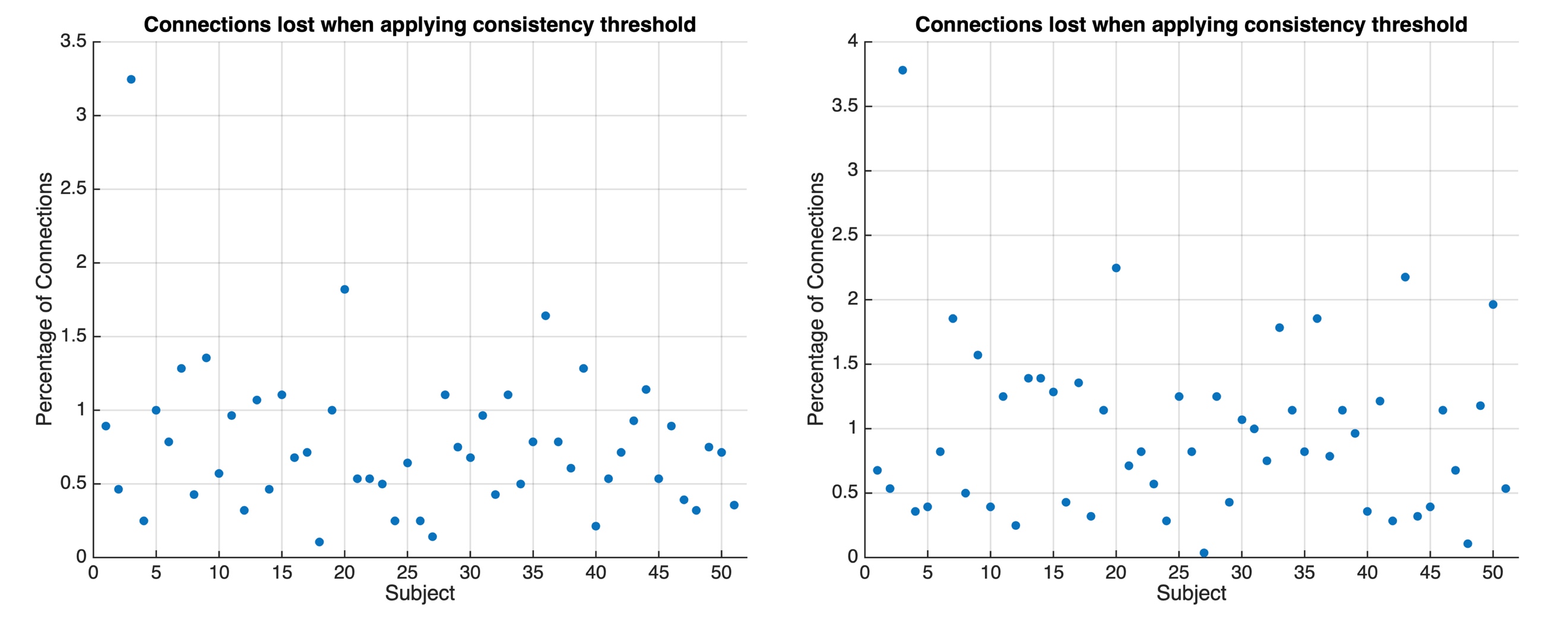
**
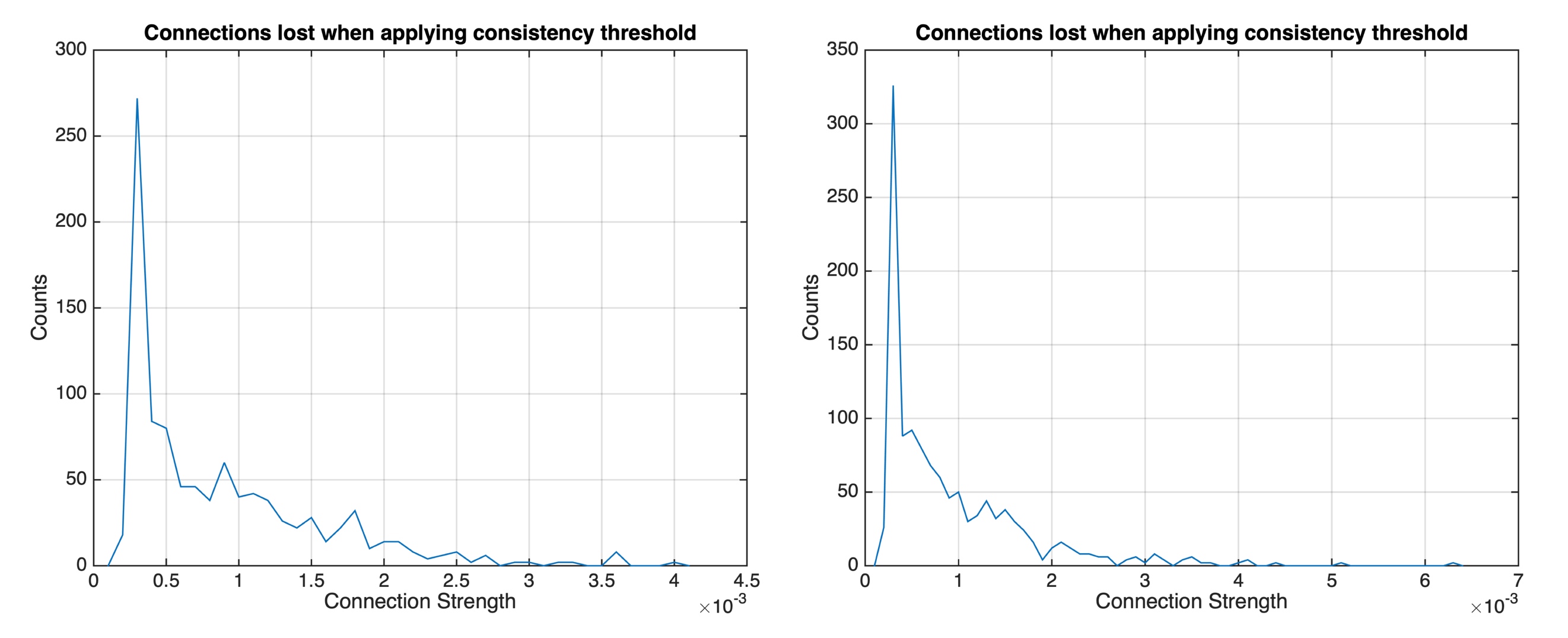
**

**Figure S3 –** Percentage of connections lost in each subject when applying consistency-based thresholding. Percentage is calculated as the proportion of connections removed in the subject SC matrix relative to the total number of connections removed in the group consistency mask. Plot on the left illustrates results for timepoint 1 and on the right, results for timepoint 2.

**Figure S4 –** Frequency distribution for the connection strength of the links removed when applying consistency-based thresholding. Plot on the left illustrates results for timepoint 1 and on the right, results for timepoint 3.


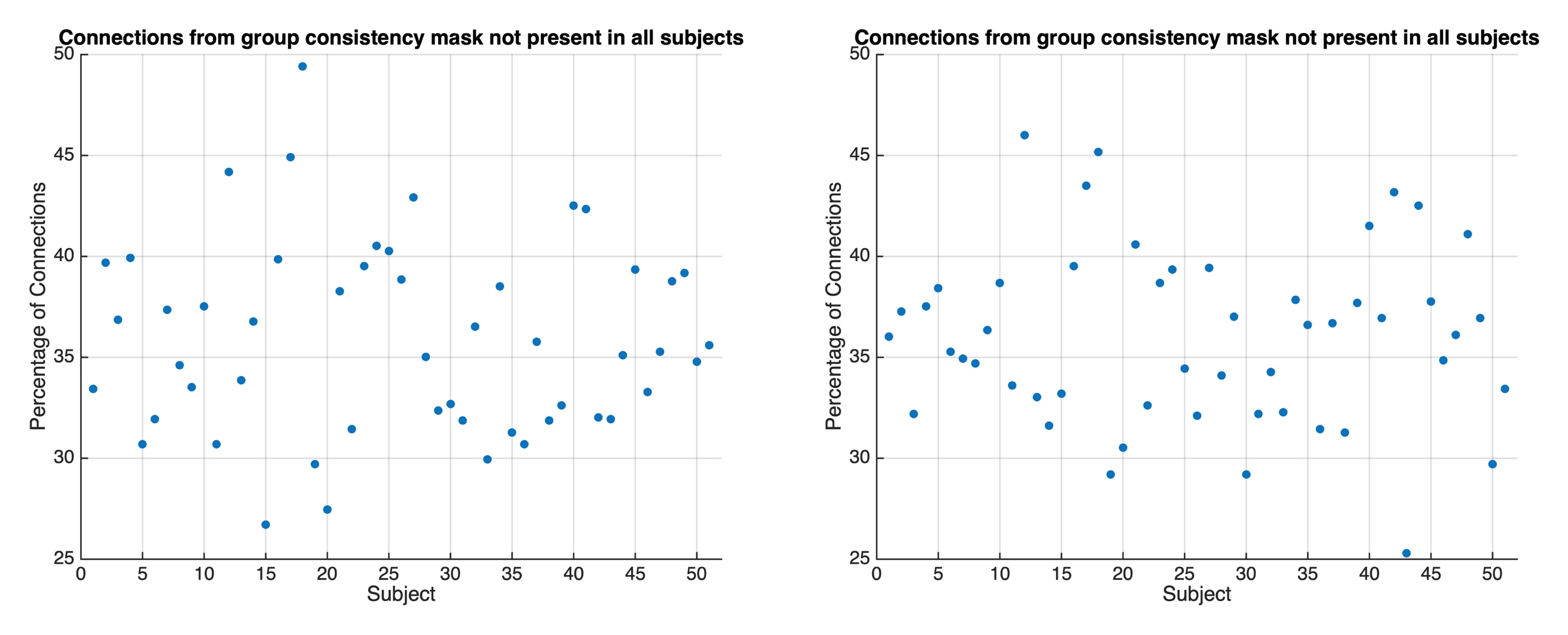


**Figure S5 –** Percentage of connections that were present in the group consistency mask but were not present in all subjects’ SC matrices. Percentage is calculated as the proportion of connections not present in the subject SC matrix relative to the total number of connections in the group consistency mask. Plot on the left illustrates results for timepoint 1 and on the right, results for timepoint 2.


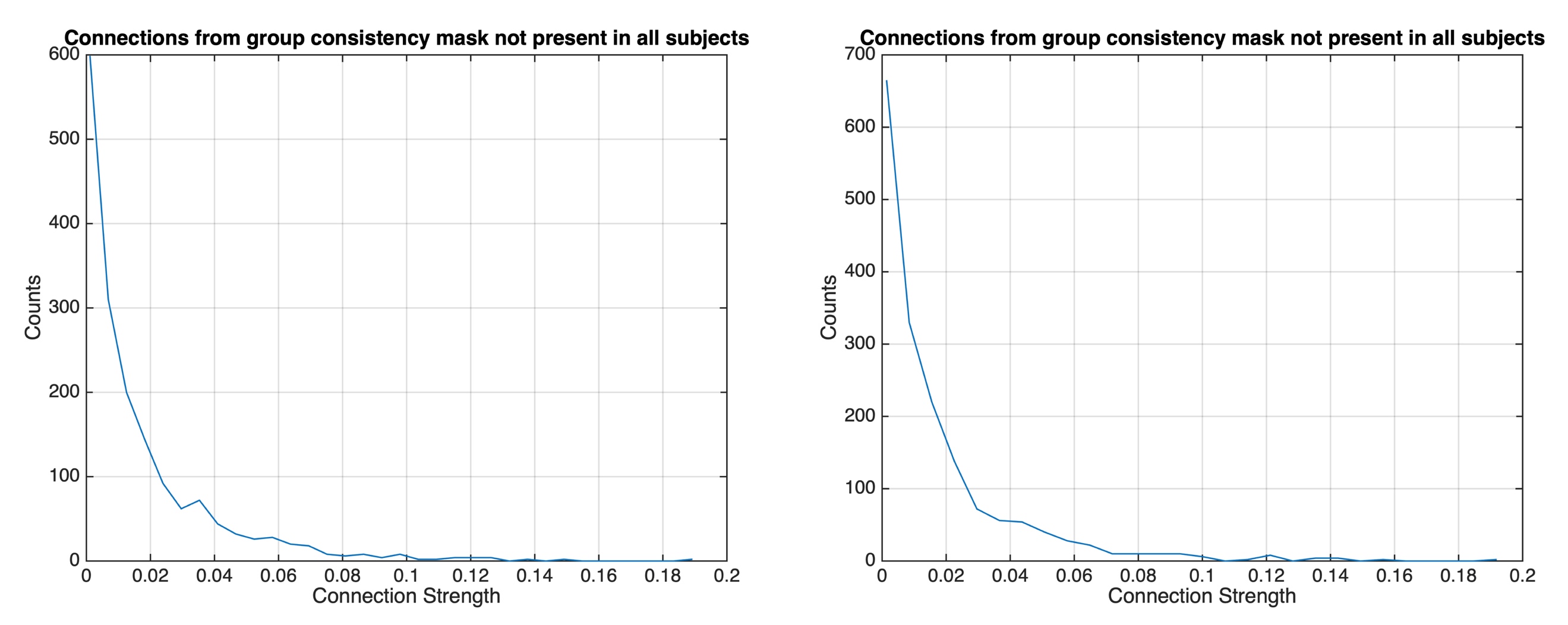


**Figure S6 –** Frequency distribution for the connection strength of the links from the group consistency mask not present in all subjects, when applying consistency-based thresholding. Plot on the left illustrates results for timepoint 1 and on the right, results for timepoint 3.


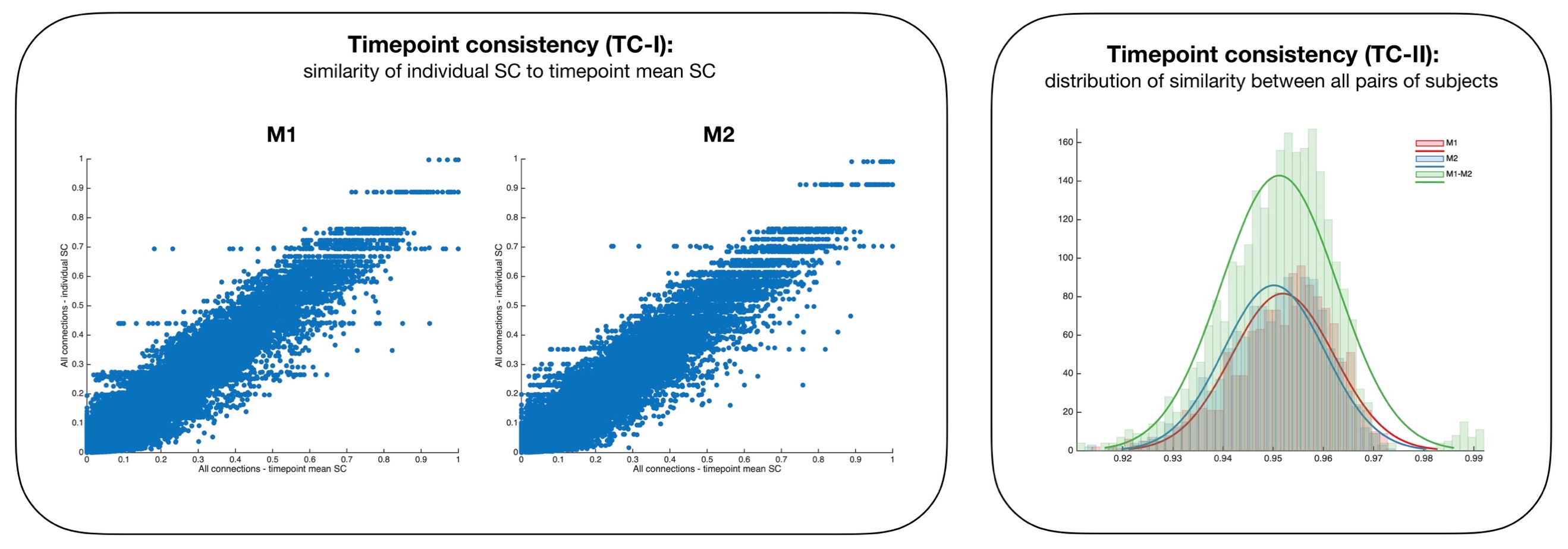


**Figure S7 –** Consistent signatures of SC for M1 and M2 timepoints. Left panel shows intra-timepoint consistency measured as the association between individual SC signatures and timepoint average SC and we can observe that the two timepoints reveal a very high level of intra-timepoint consistency (M1: 97.6%; M2: 97.5%). Right panel shows the degree of association between the signatures of SC for all pairs of subjects in the same timepoint. Once again, we notice a high level of timepoint consistency in SC (100% and 99.8% of all pairwise combinations in M1 and M2 timepoints respectively have a correlation higher than r=0.9137, with number of occurrences peaking at approximately r=0.96 for both timepoints). The overlap between the distributions of intra-timepoint consistency of both timepoints is additionally confirmed by the inter-timepoint consistency distribution (M1-M2: peak at approximately r=0.95). Taken together, these results suggest that, at a global level, the patterns of SC are highly consistent within and between timepoints, and thus potential differences due to age and sex do not have a significant impact on the estimation of SC patterns.


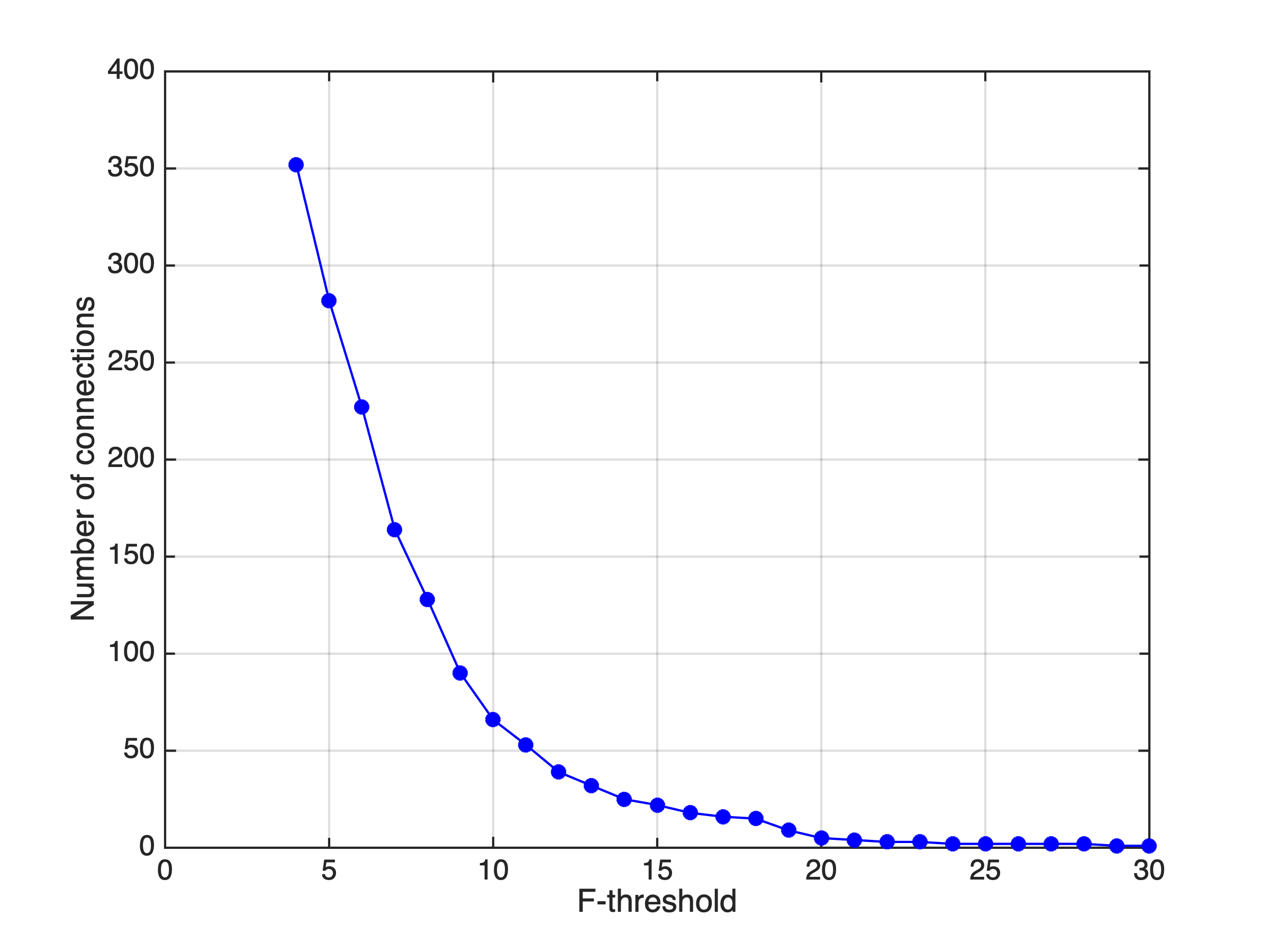

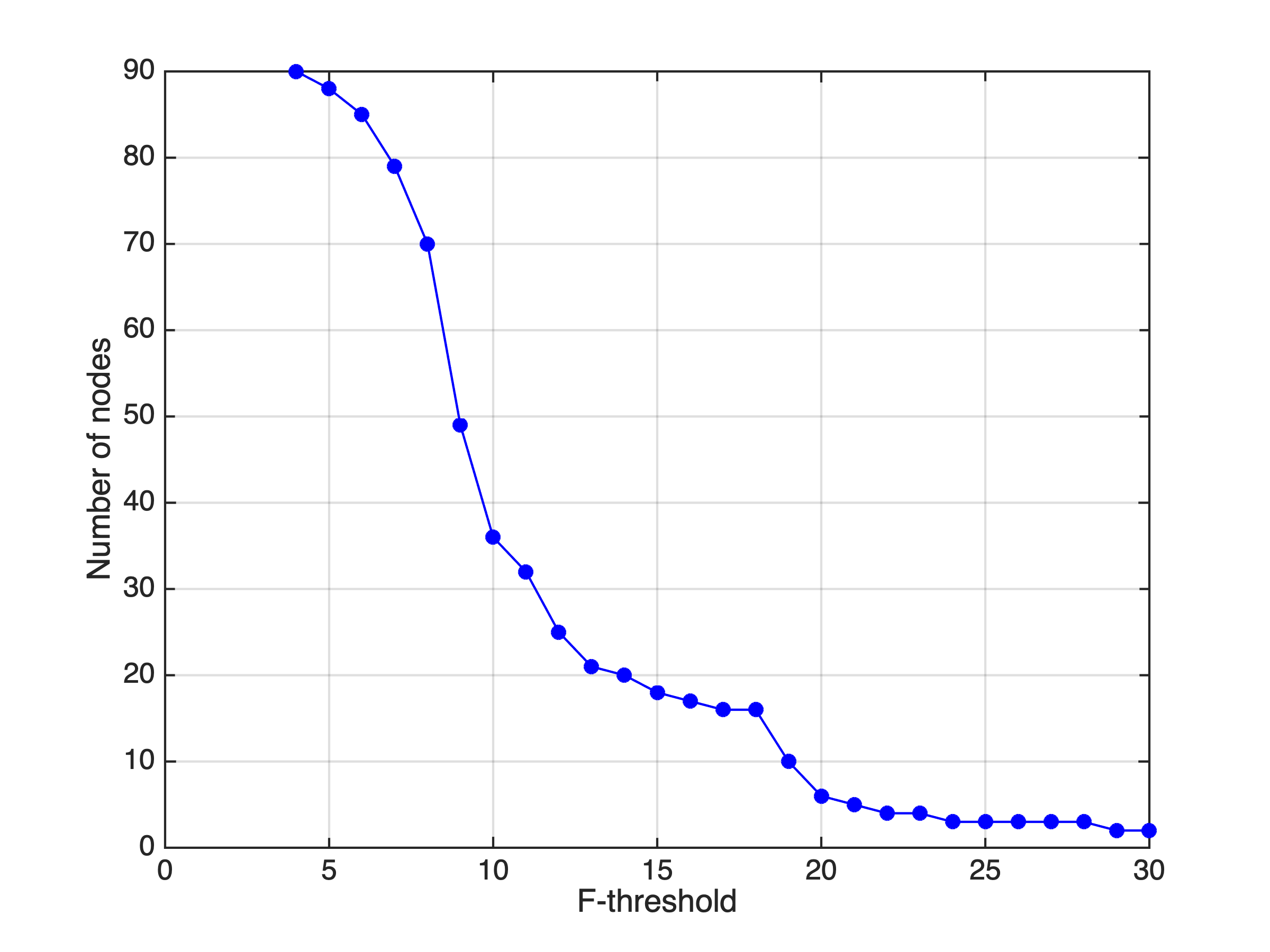


**Figure S8 -** Relationship between F-threshold and number of connections/nodes, that detected a significant component. The F-threshold used in this study (17.0) was selected based on the maximal F-threshold that detected a single component with more than two connections. This generated an NBS component with 19% nodes of the network and 16 links.


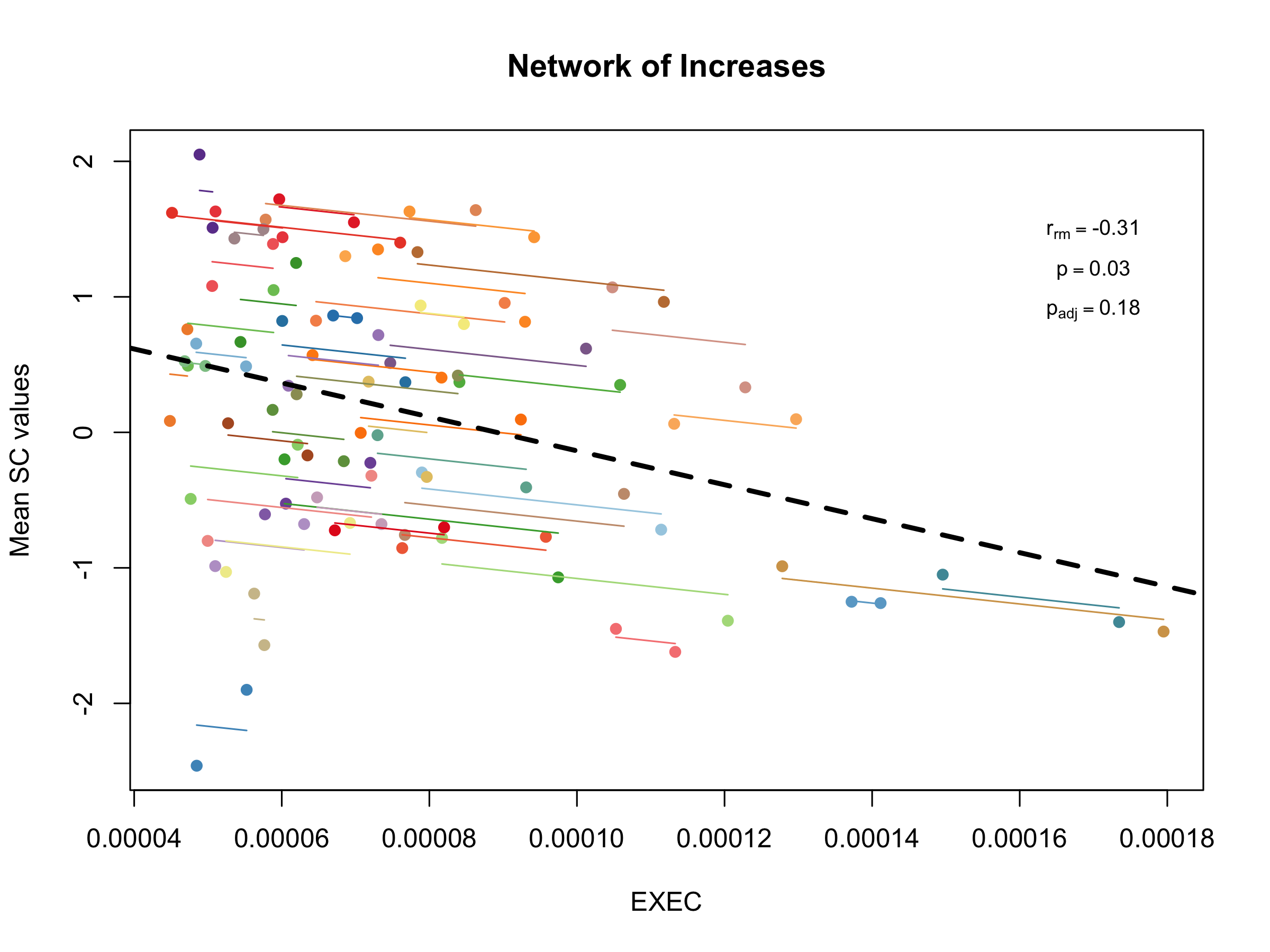


**Figure S9 –** Repeated measures correlation between mean SC values of the network with increases and mean factor scores of general cognition and executive function.

**Figure S10 -** Values of the mean number of streamlines for seed regions of the sub-network with decreases in structural connectivity. Top row shows values for timepoint M1 and bottom row shows values for timepoint M2. Seed regions are presented in rows and white matter tracts in columns.

**Figure S11 -** Values of the mean number of streamlines for seed regions of the sub-network with increases in structural connectivity. Top row shows values for timepoint M1 and bottom row shows values for timepoint M2. Seed regions are presented in rows and white matter tracts in columns.

**Supplementary Tables**

**Table S1 –** Correlations between mean SC values of sub-networks and cognitive composite dimensions (MEM and EXEC).

|  | MEM | | | EXEC | | |
| --- | --- | --- | --- | --- | --- | --- |
|  | $r_{rm}$ | p-value | p-value  (FDR corrected) | $r_{rm}$ | p-value | p-value  (FDR corrected) |
| All Connections | -0.055 | 0.70 | 0.70 | -0.11 | 0.46 | 0.70 |
| Increases | -0.27 | 0.062 | 0.19 | -0.31 | 0.030 | 0.18 |
| Decreases | 0.087 | 0.55 | 0.70 | 0.066 | 0.65 | 0.70 |

*Abbreviations:* $r_{rm}$*,* repeated measures correlation coefficient

**Table S2 –** Timepoint differences in graph theory metrics (results FDR corrected at p < 0.05).

| Network Metric | Timepoint 1 | | Timepoint 2 | | t-test | |
| --- | --- | --- | --- | --- | --- | --- |
|  | mean | SD | mean | SD | t-stat | p-value |
| Mean Connectivity | 0.013 | 0.001 | 0.013 | 0.001 | -0.17 | 0.90 |
| Degree | 1541 | 114 | 1539 | 104 | 0.082 | 0.90 |
| Connection Density | 0.19 | 0.014 | 0.19 | 0.013 | 0.14 | 0.90 |
| Global Efficiency | 0.11 | 0.006 | 0.11 | 0.007 | -0.36 | 0.90 |
| Local Efficiency | 0.11 | 0.005 | 0.11 | 0.006 | -1.23 | 0.90 |
| Clustering Coefficient ^a^ | 3.25 | 0.24 | 3.24 | 0.23 | 0.44 | 0.90 |
| Characteristic Path Length ^a^ | 1.46 | 0.026 | 1.45 | 0.029 | 3.45 | **0.009**** |
| Small-World Index | 2.22 | 0.16 | 2.23 | 0.15 | -0.13 | 0.90 |

^a^ divided by 100 random networks; * p < .05, ** p < .01, *** p < .001

**Table S3 –** Brain regions belonging to the different modules of each timepoint’s modularity community structure.

| Timepoint 1 | |  | Timepoint 2 | |
| --- | --- | --- | --- | --- |
| Module | Area |  | Module | Area |
| 1 | Precentral R |  | 1 | Precentral L |
|  | Frontal Sup R |  |  | Frontal Sup L |
|  | Frontal Sup Orb R |  |  | Frontal Sup Orb L |
|  | Frontal Mid R |  |  | Frontal Mid L |
|  | Frontal Mid Orb R |  |  | Frontal Mid Orb L |
|  | Frontal Inf Oper R |  |  | Frontal Inf Oper L |
|  | Frontal Inf Tri R |  |  | Frontal Inf Tri L |
|  | Frontal Inf Orb R |  |  | Frontal Inf Orb L |
|  | Rolandic Oper R |  |  | Rolandic Oper L |
|  | Insula R |  |  | Insula L |
|  | Postcentral R |  |  | Postcentral L |
|  | Parietal Sup R |  |  | Parietal Sup L |
|  | Parietal Inf R |  |  | Parietal Inf L |
|  | SupraMarginal R |  |  | SupraMarginal L |
|  | Angular R |  |  | Angular L |
|  | Caudate R |  |  | Putamen L |
|  | Putamen R |  |  | Pallidum L |
|  | Pallidum R |  |  | Thalamus L |
|  | Thalamus R |  |  | Heschl L |
|  | Heschl R |  |  | Temporal Sup L |
|  | Temporal Sup R |  | 2 | Hippocampus L |
| 2 | Precentral L |  |  | ParaHippocampal L |
|  | Frontal Sup L |  |  | Amygdala L |
|  | Frontal Mid L |  |  | Calcarine L |
|  | Frontal Inf Oper L |  |  | Cuneus L |
|  | Frontal Inf Tri L |  |  | Lingual L |
|  | Rolandic Oper L |  |  | Occipital Sup L |
|  | Insula L |  |  | Occipital Mid L |
|  | Postcentral L |  |  | Occipital Inf L |
|  | Parietal Sup L |  |  | Fusiform L |
|  | Parietal Inf L |  |  | Temporal Pole Sup L |
|  | SupraMarginal L |  |  | Temporal Mid L |
|  | Angular L |  |  | Temporal Pole Mid L |
|  | Heschl L |  |  | Temporal Inf L |
|  | Temporal Sup L |  | 3 | Supp Motor Area L |
| 3 | Frontal Sup Orb L |  |  | Supp Motor Area R |
|  | Frontal Mid Orb L |  |  | Cingulum Mid L |
|  | Frontal Inf Orb L |  |  | Cingulum Mid R |
|  | Olfactory L |  |  | Cingulum Post L |
|  | Olfactory R |  |  | Cingulum Post R |
|  | Frontal Sup Medial L |  |  | Precuneus L |
|  | Frontal Sup Medial R |  |  | Precuneus R |
|  | Frontal Med Orb L |  |  | Paracentral Lobule L |
|  | Frontal Med Orb R |  |  | Paracentral Lobule R |
|  | Rectus L |  | 4 | Precentral R |
|  | Rectus R |  |  | Frontal Sup R |
|  | Cingulum Ant L |  |  | Frontal Mid R |
|  | Cingulum Ant R |  |  | Frontal Inf Oper R |
|  | Caudate L |  |  | Frontal Inf Tri R |
|  | Putamen L |  |  | Rolandic Oper R |
|  | Pallidum L |  |  | Insula R |
|  | Thalamus L |  |  | Postcentral R |
| 4 | Supp Motor Area L |  |  | Parietal Sup R |
|  | Supp Motor Area R |  |  | Parietal Inf R |
|  | Cingulum Mid L |  |  | SupraMarginal R |
|  | Cingulum Mid R |  |  | Angular R |
|  | Cingulum Post L |  |  | Heschl R |
|  | Cingulum Post R |  |  | Temporal Sup R |
|  | Precuneus L |  | 5 | Frontal Sup Orb R |
|  | Precuneus R |  |  | Frontal Mid Orb R |
|  | Paracentral Lobule L |  |  | Frontal Inf Orb R |
|  | Paracentral Lobule R |  |  | Olfactory L |
| 5 | Hippocampus R |  |  | Olfactory R |
|  | ParaHippocampal R |  |  | Frontal Sup Medial L |
|  | Amygdala R |  |  | Frontal Sup Medial R |
|  | Calcarine R |  |  | Frontal Med Orb L |
|  | Cuneus R |  |  | Frontal Med Orb R |
|  | Lingual R |  |  | Rectus L |
|  | Occipital Sup R |  |  | Rectus R |
|  | Occipital Mid R |  |  | Cingulum Ant L |
|  | Occipital Inf R |  |  | Cingulum Ant R |
|  | Fusiform R |  |  | Caudate L |
|  | Temporal Pole Sup R |  |  | Caudate R |
|  | Temporal Mid R |  |  | Putamen R |
|  | Temporal Pole Mid R |  |  | Pallidum R |
|  | Temporal Inf R |  |  | Thalamus R |
| 6 | Hippocampus L |  | 6 | Hippocampus R |
|  | ParaHippocampal L |  |  | ParaHippocampal R |
|  | Amygdala L |  |  | Amygdala R |
|  | Calcarine L |  |  | Calcarine R |
|  | Cuneus L |  |  | Cuneus R |
|  | Lingual L |  |  | Lingual R |
|  | Occipital Sup L |  |  | Occipital Sup R |
|  | Occipital Mid L |  |  | Occipital Mid R |
|  | Occipital Inf L |  |  | Occipital Inf R |
|  | Fusiform L |  |  | Fusiform R |
|  | Temporal Pole Sup L |  |  | Temporal Pole Sup R |
|  | Temporal Mid L |  |  | Temporal Mid R |
|  | Temporal Pole Mid L |  |  | Temporal Pole Mid R |
|  | Temporal Inf L |  |  | Temporal Inf R |
